# Supplementary material for: Surface Microbiome Profiling of Dental Elevators Using Third‐Generation Sequencing: Implications for Infection Control in Dental Practice
Source: Microbiologyopen. 2025 Dec 8;14(6):e70178. doi: 10.1002/mbo3.70178 (PMC12685768; doi:10.1002/mbo3.70178)
Supplement: Supplementary file 1 — customer_backup. [file MBO3-14-e70178-s001.zip › customer_backup/customer_backup/taxa_summary/krona/samples/M04.Krona.html]

Javascript must be enabled to view this page.

members
magnitude
magnitudeUnassigned

M04.krona

64805

64793

67

67

67

67

67

25

13

10

6

13

6470

4

4

1

1

1

3

2

2

1

1

109

109

109

109

109

1512

835

6

6

6

378

251

48

15

10

86

57

35

4

4

5

5

40

40

48

30

9

9

30

30

451

49

49

389

389

13

13

677

263

263

13

250

414

1

1

413

413

1254

56

56

56

56

143

29

29

29

22

22

22

1

1

1

91

91

91

320

6

6

6

11

11

11

30

30

30

6

6

6

231

231

231

36

36

36

1

1

1

1

734

48

43

43

5

5

158

158

2

13

143

327

327

327

201

19

1

18

148

148

34

15

19

3563

14

14

3

3

11

2

9

15

15

15

15

1

1

1

1

36

36

1

1

35

1

3

6

1

19

5

321

21

7

7

12

12

2

2

300

10

3

7

5

5

268

10

5

1

47

100

2

9

2

10

5

42

15

19

1

17

3

14

18

18

1

1

3

3

3

2

1

2

2

5

5

1

1

3

3

12

12

12

1

11

9

1

1

1

6

6

6

2

1

1

1

1

27

27

8

8

19

1

1

3

13

1

246

119

23

23

94

94

2

2

15

2

2

13

13

1

1

1

111

5

5

13

13

93

93

22

22

6

1

5

7

1

3

3

1

1

3

3

1

1

4

4

7

4

4

4

3

3

3

2835

2321

1

1

11

11

3

3

282

282

343

343

1681

2

1160

42

350

127

35

5

5

4

4

9

9

5

5

12

12

257

236

236

21

21

192

4

4

6

6

8

8

9

9

4

4

6

6

141

1

27

113

1

1

9

9

4

4

30

24

24

6

6

28

28

28

28

6

20

2

45

44

44

44

6

6

38

38

1

1

1

1

1

592

592

592

592

592

592

32884

32884

32884

32877

32870

32870

7

6

1

7

7

7

49

49

49

35

35

1

34

14

14

14

45

24

24

21

21

21

3

3

3

21

21

7

7

7

14

14

14

8

2

1

1

1

1

1

1

1

1

6

4

4

4

4

2

1

1

1

1

1

1

13

13

13

13

13

4

9

34

2

2

2

2

2

31

31

31

2

2

29

23

1

5

1

1

1

1

1

31

31

31

31

31

31

1915

24

24

24

24

24

27

9

9

9

9

7

7

7

7

11

11

11

11

1864

1864

1294

1294

1294

8

8

8

53

53

53

38

38

38

257

242

242

15

15

84

67

67

17

17

130

130

130

542

216

7

7

7

7

209

103

84

77

7

1

1

18

18

106

39

39

1

1

66

66

109

15

15

15

10

5

3

3

3

3

91

91

36

17

19

13

13

27

3

15

9

15

15

58

58

58

58

6

37

6

9

15

15

15

15

15

144

144

21

21

21

117

117

117

6

6

6

3

3

3

3

3

3

495

24

24

24

24

24

163

163

163

10

10

86

86

47

8

39

20

9

11

308

308

308

217

12

100

105

70

70

7

7

14

14

443

111

111

111

111

111

332

138

130

130

84

46

8

8

8

123

68

6

6

15

15

47

25

22

46

46

46

9

9

1

8

71

71

14

14

57

3

54

39

39

39

16

16

16

23

7

7

16

2

14

133

90

13

13

4

4

9

9

77

51

51

51

18

18

18

8

8

8

43

35

35

12

12

23

23

8

8

8

8

14745

9081

112

112

88

88

24

24

321

32

32

32

244

12

12

10

10

222

222

45

45

45

7

6

6

6

1

1

1

64

64

64

3

61

30

30

30

30

4896

4896

8

8

266

21

7

58

180

69

69

133

133

5

5

5

5

22

6

16

755

461

121

173

3549

6

297

71

7

7

766

905

1169

82

1

27

202

2

7

84

1

83

17

8

8

8

9

9

9

98

98

37

37

13

13

29

22

7

18

4

14

1

1

2047

186

15

15

11

11

27

27

133

29

104

3

3

3

9

9

9

1

1

1

133

17

17

64

14

6

44

52

52

1

1

1

83

11

3

8

9

9

63

18

34

11

7

7

7

40

22

21

1

18

18

495

3

3

36

36

1

1

134

134

12

12

248

44

39

123

4

38

6

6

55

6

23

14

10

1

1

230

10

3

7

24

24

94

94

35

35

1

1

66

21

45

88

1

1

53

53

34

34

771

312

312

8

8

2

2

18

18

364

32

15

13

139

28

31

56

9

18

23

27

27

14

3

2

9

26

26

213

213

95

8

5

50

32

33

7

26

27

1

26

13

13

45

45

7

7

7

7

6

6

6

6

209

10

10

10

150

150

150

4

4

4

22

22

22

23

23

23

26

26

22

22

4

4

1028

1028

1

1

9

9

13

13

33

4

11

18

45

21

24

9

9

867

20

313

534

51

51

5664

16

16

6

6

10

10

334

1

1

1

3

3

3

78

11

11

5

5

6

6

56

56

22

22

20

2

16

1

1

15

15

1

1

1

213

213

213

3289

29

29

29

9

9

9

7

7

7

49

49

49

1688

494

494

464

9

8

42

405

730

16

26

52

41

49

1

15

28

40

7

409

11

35

158

6

6

91

11

80

39

39

22

22

61

37

37

6

6

18

18

5

4

4

1

1

72

72

72

1208

31

2

29

25

25

13

13

32

32

84

84

73

39

34

118

98

20

540

87

453

59

59

1

1

11

11

221

221

3

3

3

34

30

18

17

1

12

12

4

4

4

17

17

11

11

6

6

5

5

5

5

1

1

1

1

86

6

6

6

80

80

80

1800

385

64

64

34

34

287

287

1415

4

4

3

3

38

21

5

12

1370

387

3

5

502

41

46

1

29

356

12

12

12

12

6

6

6

6

64

13

13

13

25

25

1

19

5

8

8

8

18

2

1

1

16

1

6

9

613

436

436

436

27

27

41

41

82

82

30

30

5

5

33

33

218

168

39

11

177

9

9

9

9

75

44

44

44

17

7

4

3

10

10

14

14

14

17

17

4

4

13

13

76

76

76

76

47

47

47

47

47

33

8

6

129

18

11

11

11

11

7

7

7

7

13

13

5

5

5

8

8

8

29

1

1

1

1

20

20

20

20

6

6

6

6

2

2

2

2

69

69

69

69

69

3403

909

1

1

1

1

3

3

3

2

1

676

676

7

7

2

2

9

4

3

2

53

17

36

12

12

25

22

3

14

2

2

5

5

5

4

1

38

37

1

23

1

22

63

63

2

1

1

2

2

1

1

58

7

50

1

65

65

2

2

1

1

1

1

20

4

16

272

5

36

1

1

176

33

1

19

1

1

74

3

1

1

1

1

1

1

70

3

3

19

19

1

1

47

45

2

1

1

1

24

24

1

1

20

17

1

1

1

3

3

18

3

3

3

9

9

9

6

6

6

12

12

12

12

99

80

1

1

1

1

17

17

1

1

17

17

2

2

12

2

9

1

29

1

28

7

7

7

8

2

1

1

6

6

4

4

4

2

1

1

1

1

1

1

2392

700

498

498

33

7

2

277

18

23

18

83

7

10

7

13

202

85

85

21

21

73

73

1

1

22

1

21

9

9

9

9

166

64

62

62

1

1

1

1

102

19

19

3

3

25

25

28

28

13

13

10

10

3

1

2

1

1

298

298

9

9

8

8

241

21

25

161

1

9

1

14

2

1

6

12

12

28

23

5

24

24

5

5

1

1

18

4

14

559

559

559

558

1

5

1

1

1

4

4

4

618

6

6

5

1

31

15

15

1

1

15

15

160

155

40

32

2

66

15

5

5

1

1

1

419

2

2

7

6

1

2

1

1

230

2

86

123

19

18

18

5

5

106

106

21

20

1

5

1

4

14

14

9

2

7

1

1

1

6

6

6

6

7

7

7

7

8

8

8

8

8

67

67

67

62

62

5

5

27

27

27

27

27

27

27

27

27

27

1

26

223

223

223

223

223

113

110

1418

1418

1034

9

9

9

1005

31

1

29

1

3

3

13

13

524

17

30

457

16

1

3

160

96

64

1

1

49

15

34

22

22

106

84

22

96

96

20

20

9

11

5

2

1

1

1

1

3

3

3

135

18

16

16

2

2

1

1

1

27

27

4

8

3

12

7

1

1

1

1

5

2

2

1

64

1

1

37

5

32

1

1

25

13

6

6

18

5

5

3

3

10

10

27

26

3

3

17

17

6

6

1

1

1

217

14

10

10

4

4

9

9

9

14

12

12

2

2

1

1

1

32

29

29

3

3

147

53

37

16

15

10

5

9

9

70

3

5

2

3

22

8

27

374

316

23

23

12

12

11

11

293

277

277

277

16

16

16

58

43

43

14

14

24

24

5

1

4

15

15

15

15

6

6

6

6

6

6

12

4

4

4

4

4

4

7

7

7

7

7

7

1

1

1

1

1

1
